# Exploration of ‘generational’ peer-led CPR training in the Australian community using blended learning approaches: a pilot randomised controlled trial

**DOI:** 10.1016/j.resplu.2025.101190

**Published:** 2025-12-15

**Authors:** Jeremy Pallas, Mark Miller, Shaun Hicks, Phillip Newton, Ginger Chu, John Paul Smiles, Michael Zhang

**Affiliations:** a School of Nursing and Midwifery, University of Newcastle, Australia; b Emergency Department, John Hunter Hospital, Hunter Heart Safe, Australia; c Nephrology Department, John Hunter Hospital, Australia; d Asthma and Breathing Program, Hunter Medical Research Institute, Australia; e Emergency Department, John Hunter Hospital, Australia

**Keywords:** Cardiopulmonary resuscitation, Peer-led training, Blended learning, Cardiac arrest

## Abstract

**Aim:**

A pilot randomised controlled trial to assess the feasibility and relative effectiveness of a community CPR train-the-trainer model using either a traditional face-to-face or blended learning approach (video supported face-to-face training with the provision of a multimodal ‘CPR lesson card’ containing visual prompts and a QR linked training video). A ‘generational’ recruitment strategy was used to evaluate knowledge degradation across a series of peer-led training episodes.

**Methods:**

Participants (*n* = 155) were community volunteers aged 18–85 years with no recent CPR training. Groups were randomised to either face-to-face (control) or blended training (intervention) groups. The first participant in each stream (Generation one) received professional training and subsequently taught the next generation, continuing up to four generations. Progression required passing a simulated cardiac arrest assessment against a critical item checklist including a QCPR score ≥50.

**Results:**

In total, 115 CPR assessments were conducted (57 intervention, 58 control) following episodes of intergenerational peer-led training. Pass rates were 96.5 % (55/57, 95 % CI: 87.9–99.6 %) in the intervention group and 75.9 % (44/58, 95 % CI: 62.8–86.1 %) in the control group (Fisher’s Exact *p* ≤ 0.05). The combined pass rate between both groups was 86 %, supporting feasibility of peer-led CPR training.

**Conclusion:**

Peer-to-peer CPR training in the community is feasible through several generations of knowledge transfer. The use of a simple multimodal training aid appears to enhance performance beyond the first generation and may provide a scalable, cost-effective adjunct to traditional CPR training.

## Introduction

Survival rates for out-of-hospital cardiac arrest (OHCA) are generally low with wide international variation.^1^ The poor outcomes associated with OHCA have remained mostly static in the last three decades.^2^ The rate of survival to discharge after cardiac arrest in the Australian community is approximately 4 % for all cardiac arrests and 10.9 % for those in whom Emergency Medical Services (EMS) attempted resuscitation.^3^ It has been identified that there is a substantial gap between the large disease burden associated with OHCA and the modest investment in prevention and treatment^4^ – a factor which supports the need for novel and efficient strategies to improve outcomes.

Early, high-quality CPR is directly related to neurologically intact survival from OHCA.^2^, ^5^ Community CPR training has been correlated with improved bystander CPR rates,^6^, ^7^, ^8^ a factor that has been linked to increased survival from OHCA.^5^ Despite the availability of CPR training in Australia, bystander CPR rates remain unacceptably low with New South Wales (NSW) Ambulance data indicating bystander CPR is only attempted in 34 % of OHCA^10^ – below the Australian average of 38.2 %.^3^

In Australia, CPR training is primarily provided by ‘professional’ trainers from a variety of backgrounds.^9^ Zhang et al found that peer led training was associated with improvement in resuscitation skills and knowledge when compared to professionally led training.^11^ Existing studies investigating peer-led CPR training have primarily focused on school students and healthcare professionals/trainees.^12^, ^13^ Outcomes in these cohorts are broadly favourable with peer trainers demonstrating similar effectiveness and learner satisfaction compared to professional instructors.^14^, ^15^ While community-based peer-led CPR initiatives have been described,^16^ only one study^17^ directed investigation beyond the single tier or ‘generation’ of peer training. Where a basic ‘peer-to-peer’ model may support scaling of community CPR training, a multi-generational approach could foreseeably compound this scaling effort. Additionally, a peer led CPR training model in the community may reduced dependency on the finite professional training workforce.

In response to these knowledge gaps, this study aims to assess the feasibility and relative effectiveness of a community CPR train-the-trainer model using either a traditional face-to-face or blended learning approach combining face-to-face techniques, instructional videos, and a simple multimodal training aid.

## Methods

### Trial design

A pilot randomised controlled trial using a generational training approach to compare the control group (face-to-face training) and intervention group (blended training). A ‘generational’ recruitment strategy was employed to evaluate knowledge degradation across a series of peer-led training episodes. This study was approved by the Hunter New England Human Research Ethics Committee prior to recruitment. No prospective clinical trial registration was attended. The full trial protocol is available as an online supplement.

### Participants and recruitment

All participants were community-based volunteers from the Newcastle and Lake Macquarie areas in New South Wales, Australia. Participants were recruited through social media advertisement on the Hunter Heart Safe (a CPR training not for profit organisation) Facebook page to community groups in the greater Newcastle area. Recruitment occurred between January 2023 and December 2024. Eligibility criteria were:

1. Aged between 18 and 85 years
2. No physical or medical limitations to performing chest compressions for one minute
3. Not attended CPR training in the past five years

### Randomisation and blinding

**Fig. 1:**
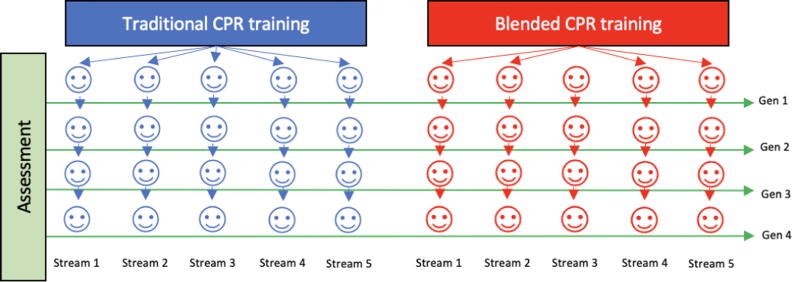
**Graphical representation of participant flow in the groups (Gen = generation or position within the linear progression through the individual stream)**.

Participating groups were randomised using the electronic resource available at https://www.randomizer.org. Study packs were compiled before commencing recruitment, with group assignment to either control or intervention being concealed in opaque envelopes that were opened immediately prior to the commencement of each session. Once the larger group had been randomised, individual participants were further randomised into five ‘streams’ of four participants. From the larger group of 20, each participant was randomly allocated a coloured and numbered paper tag denoting their place in the training flow of the session. The first participant in each stream (generation one) received either traditional face-to-face CPR training OR a blended training session based on group randomisation. The three participants following from the initial trainee in each ‘stream’ are referred to as subsequent ‘generations’, i.e. the second person in a stream becomes the second generation of that stream, the fourth person in sequence is the fourth generation, etc. A visual representation of the participant flow is presented in Fig. 1**.** The nature of the intervention did not allow for participants or the study team to be blinded to the group allocation after randomization had occurred.

### Sample size

A convenience sample was employed aiming to recruit 160 total participants across eight groups of 20 (group size determined by generational methodology). For pragmatic reasons, no demographic data were prospectively collected on participants in this trial.

### Trial Setting

All recruitment, teaching and data collection attended during this study took place in public community meeting sites including community halls, a restaurant ballroom and a residential movie theatre.

### Interventions

#### Training approach

Groups of 20 participants were recruited and randomised into five streams of four participant ‘generations’. The first-generation participants from all five streams were trained together at the commencement of each session (away from the rest of the group). In both the control and intervention groups this training took approximately 30 min, was facilitated by the principal investigator and focused on basic compression only CPR skills. Intervention group training was delivered using purpose developed training videos as a foundation. These videos were also provided as a QR coded link in the CPR lesson cards that were provided to the intervention group as training aids. In the control group, no videos or other cognitive aids were used with all teaching coming directly from the principal investigator. All training sessions included hands on practise and demonstration using Laerdal resuscitation mannequins.

Following initial training, the first generation from each stream taught the lesson they had learned (one-on-one) to the second generation. No instructions on how to teach were provided, but all participants had access to resuscitation mannequins and automated external defibrillator (AED) trainers. If successfully taught, the second generation was tasked with teaching the third (over a period of 20 min), and so on until either the message failed to be delivered appropriately or all four generations within a single stream had been successfully taught. All the peer training sessions for a given group occurred in a single three-hour session on the same day.

#### Multimodal training aid – ‘CPR lesson cards’

**Fig. 2:**
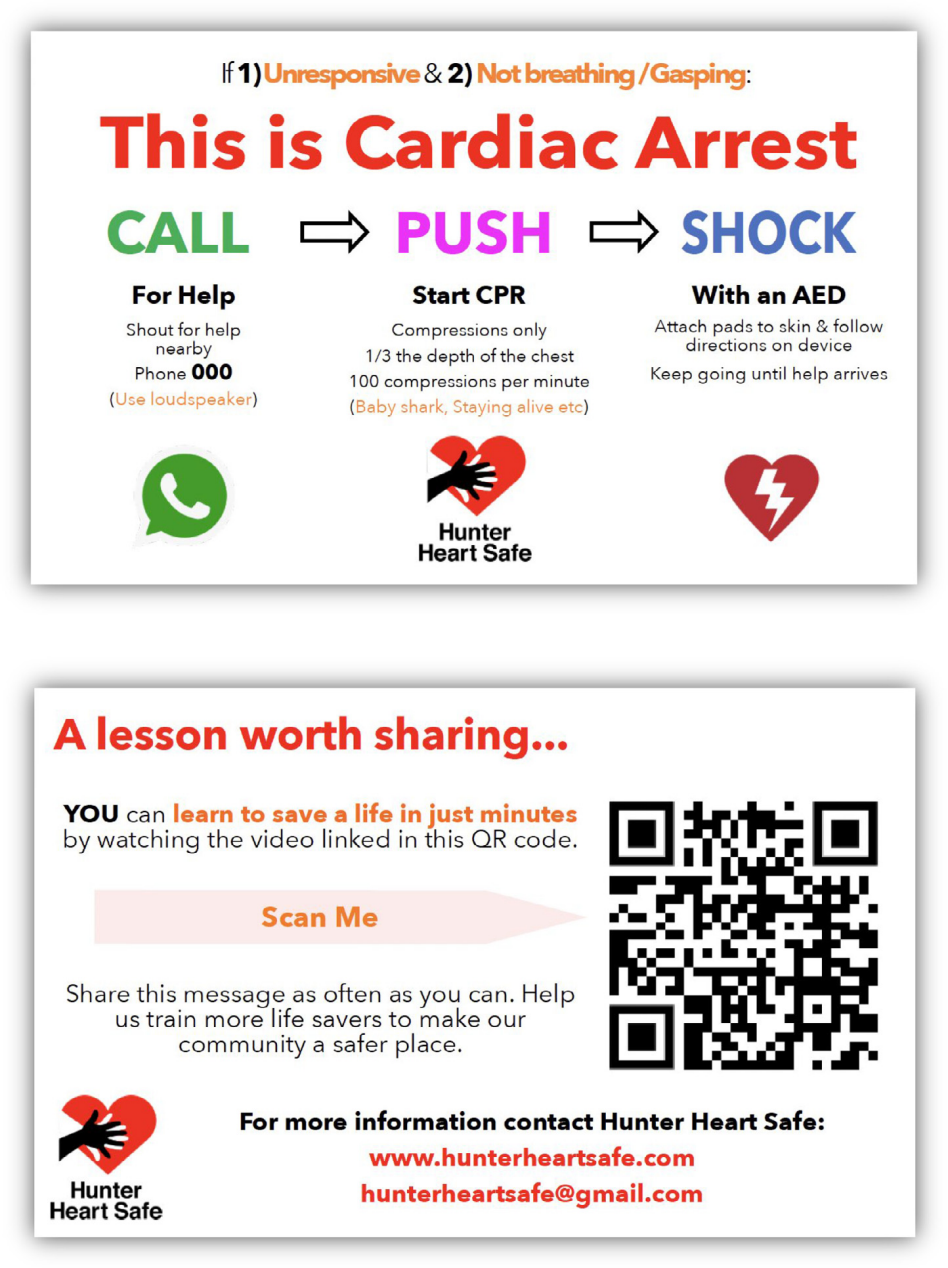
**Both sides of the ‘CPR lesson cards’ provided to participants in the intervention group of this study with a QR coded link to the following video**https://youtu.be/wHPJ2Zbsv6c?si=p_p8UDp6YlU8kneT.

Within the intervention groups, a multimodal training aid ‘CPR Lesson card’ (Fig. 2) was introduced as an adjunct to the delivery of a blended learning style CPR lesson. The card was a double-sided business card with a basic CPR sequence on one side and a QR coded link to a video-based CPR lesson on the other side. The embedded QR code linked directly to a CPR training video developed by the research team. This video was used as both the basis for the initial instruction provided to the first generation of the intervention group, and as a teaching aid to support their subsequent peer-led training. No cognitive aids were provided to the control group who received standard face to face training.

#### Intergenerational assessment

For one generation to progress from learning to teaching, they must successfully complete an intergenerational assessment against a seven point ‘critical item checklist’ (available as an online supplement) that was developed for this trial aggregating recognised compression only CPR targets. The first six items of the checklist were simple binary actions like checking for consciousness or correctly using an AED, while the final item required participants to physically perform CPR on mannequin. If participants were able to successfully perform all seven tasks on the checklist (including a QCPR score >50), they were graded as a pass. If they were unsuccessful in any area of the checklist they failed the assessment.

While completing the critical item checklist, all participants were required to perform one minute of compression-only CPR on a Laerdal resuscitation mannequin to achieve a QCPR score. The effectiveness of chest compressions is measured by the mannequin, transmitted to a smartphone-based app (Laerdal QCPR) and represented as a percentage score of effective compressions. A pragmatic decision was made to include a QCPR score of 50/100 as the passing grade. While this score may be considered too low to reflect maximal chest compression quality, given the focus of this trial on generational teaching, we deemed a score of 50/100 reflected a suitable lowest limit for a passing attempt. All assessments were facilitated by volunteers through the Hunter Heart Safe organisation (a mix of Doctors, Nurses and Paramedics).

The first generation was exempt from this assessment as the efficacy of the initial professional CPR training sessions was not the focus of this trial. If any member of the stream failed the assessment, data collection for that stream ended, and all remaining members of that stream were deemed to have completed their participation in the trial.

### Outcomes

The primary outcome was the feasibility of peer-led CPR training as demonstrated by the total proportion of recruited participants who were successfully trained, measured by practical CPR assessments using a ‘critical item checklist’ (available as an online supplement). The secondary outcome was the effectiveness of a blended learning approach compared to face-to-face training for facilitating peer-led CPR training measured by practical CPR assessments against the same ‘critical item checklist’.

## Results

**Fig. 3:**
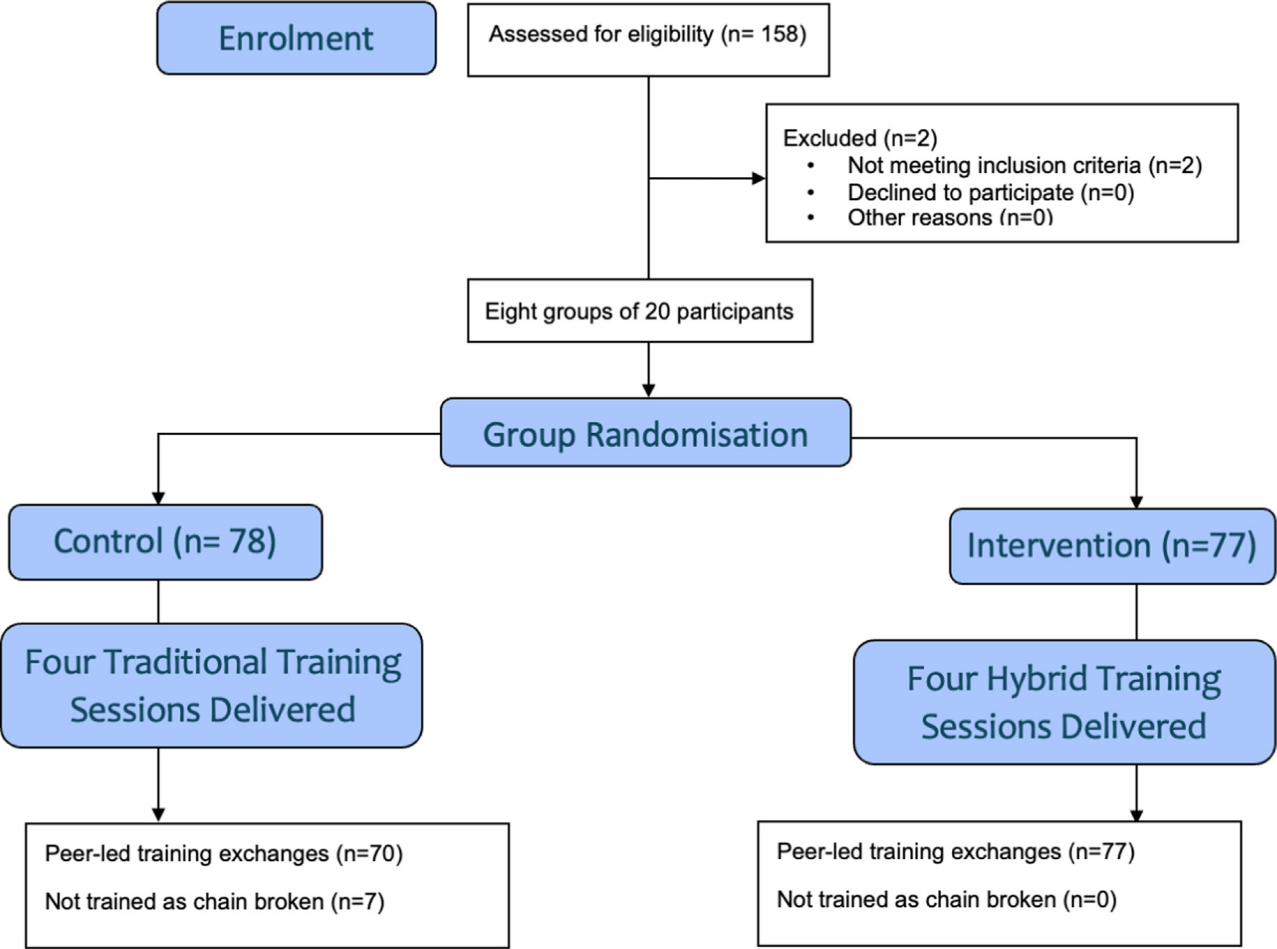
**CONSORT flow diagram**.

In total 155 participants were recruited across eight sessions with 77 participants in four intervention groups and 78 participants in four control groups. Two participants were excluded at the point of initial screening as they did not meet the inclusion criteria (one having a pre-existing injury and the other being over the maximum age cutoff). A table containing the full study results is available an online supplementary item (Fig. 3).

### Peer-led CPR training feasibility

**Fig. 4:**
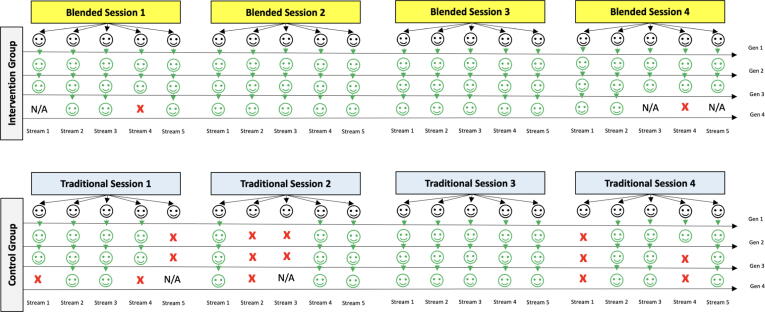
**Graphical representation of the successful assessments across the patient cohort (Green = successful assessment, red = unsuccessful assessment, N/A = no available participant)**. (For interpretation of the references to colour in this figure legend, the reader is referred to the web version of this article.)

Because generation one participants were exempt from assessment, there were 57 possible inter-generational CPR assessments that could be passed in the intervention group and 58 in the control group (accounting for slight under-recruitment in the later generations of each group). The recruitment and outcomes of this study are visually represented in Fig. 4.

In the intervention group, 55/57 (96.5 %, 95 % CI: 87.9–99.6 %) assessments resulted in a passing grade. In the control group, 44/58 (75.9 %, 95 % CI: 62.8–86.1 %) assessments resulted in a passing grade. The higher pass rate in the intervention group was statistically significant (Fisher's Exact *p* ≤ 0.05). In total, 99/115 (86 %) possible assessments were passed between both arms of the study. While no a priori feasibility threshold was set prior to recruitment, performance in both the intervention and control groups was sufficient to support the overall feasibility of a peer-led approach to CPR training.

Of the 5 intervention groups that did not progress to a successful fourth generation, only two of these streams had fourth generation participants available, with the remaining three streams having a total of three (not four) members secondary to recruitment challenges. These three groups demonstrated 100 % successful transmission between available generations, with transmission between the third and fourth generation participants unable to be assessed. In the control group there were no instances where the progress of an individual stream interrupted by under-recruitment with all early breaks in the ‘training chain’ in this group resulting from assessment failures.

## Discussion

This trial hypothesised that peer-led training would be feasible and effective following both initial training approaches, with superior effectiveness predicted in the intervention group owing to the blended learning approach and the introduction of multimodal training aids. The experimental design intended to test the extent to which peer-led training sessions can chain together without assistance from a professional trainer. In a real-world application, this flow might represent a community member attending CPR training before returning home to teach a family member, who subsequently teaches a friend, who then teaches a classmate and so on.

Within the intervention group, the integrity of the peer-led training was preserved in all groups through the first three generations. This means that from a group of 20 initial trainees, a further 40 people were trained effectively without the involvement of a professional CPR instructor. Further, when the message was peer-taught for a third time, it was effectively communicated in 15/17 (88 %) possible interactions. In the two failed exchanges, each trainee failed to call for help and one failed to cheque for breathing – However, both trainees correctly commenced CPR, achieving QCPR scores of 99 and 44 respectively.

Positive outcomes were also identified in the control group with peer-led training being successful in 75.9 % of cases. The relative success of peer-led training sessions in this group reinforces the general feasibility of the peer-led CPR training concept. The positive outcomes following peer-led CPR training in this trial add to the foundation of efficacy established by Wik et al.^17^ who identified community-based peer trainers had similar training outcomes when compared to professional instructors through both first- and second-generation encounters.

In the control group there were four failed assessments immediately following the first attempt at peer-led training. It is possible the absence of CPR lesson cards in this stream made it more difficult to carry forward the training message between generations. Alternatively, the inclusion of multimodal teaching in the intervention group may have increased the effectiveness of their initial training. Given the lack of first-generation assessment in this study, there is not enough data to clearly differentiate between these possibilities. The utility of cognitive aids for peer-trainers seems supported.

In relation to the CPR lesson cards, it was anticipated that most peer-trainers would choose to use the QR linked video as a foundation for their own teaching through the generations. However, it was directly observed that the cards were often used as simple visual aids to prompt their own live presentation. While it was intended for all participants in the intervention group to have access to a blended learning approach (inclusive of videos), this was only consistently applied for the initial training of the first-generation participants – with peer trainers alternating between a true blended learning approach and more conventional training based around a static cognitive aid. As such, the relative performance impact of the videos cannot be separated from the simple printed card.

The traditional ‘instructor-centric’ approach to community CPR training is limited by the availability of professional trainers and cost. Peer-led community CPR training may have the potential to increase training rates significantly. Such an approach can reasonably be associated with benefits relating to scaling and cost, and can be easily added into existing CPR training programmes. Trainees are likely to be able to successfully teach the key messages that enable a meaningful cardiac arrest response onto their family and friends. The positive outcome in this study may suggest a viable role for a multi-modal training aid to support peer led training beyond the initial point of professional training. However, it is noted that a larger trial would be beneficial to further substantiate the impact of such an intervention.

### Limitations

Firstly, the generalisability of this trial is limited by its small sample size (155 participants), and the use of a convenience sampling strategy. Most sessions (6/8) were recruited from over 55’s communities or retirement-focused groups, introducing an age bias. Additionally, the absence of demographic data prevents analysis of participant-level factors impacting performance. Secondly, although participants self-assessed physical limitations, some underestimated the effort required for chest compressions, occasionally affecting CPR quality. Additionally, cognitive impairment was not screened, resulting in two participants reporting post-training difficulty, likely contributing a minor negative impact on outcomes. Finally, excluding the first generation from assessment limits the ability to separate peer-to-peer loss from instructor variability in early failure. The pragmatic CPR pass threshold (QCPR ≥ 50) may not reflect high-quality performance and could reduce generalisability. Additionally, conducting multiple episodes of peer-led training within 3 h of the primary professional training limits ecological validity.

Owing to the educational nature of this trial and the lack of patient level clinical outcomes, this study was not prospectively registered as a clinical trial which limits methodological transparency. Accordingly, the full study protocol is available as an online supplement to this manuscript.

Future studies should validate these findings through larger scale randomised controlled trials to better assess the effectiveness of the peer-led CPR training. Further research should also examine knowledge retention and CPR performance over time to capture the long-term temporal decay associated with this approach.

## Conclusion

These findings support the hypothesis that community peer-led CPR training is feasible through several generations of knowledge transfer. The provision of a simple multimodal training aid with embedded instructional videos appears to improve the effectiveness of peer-led CPR training beyond the first generation, a finding which warrants confirmation in a larger trial. This approach may offer efficiencies for training the wider community in cardiac arrest care.

## Disclosures

No author on this trial has any financial (or otherwise) conflict of interest to disclose related to this trial.

## Open Science

The full trial protocol is available as an online supplementary item. The final data table containing non-identified trial results is available as an online supplementary item. Additional requests for data sharing should be directed to the corresponding author.

## CRediT authorship contribution statement

**Jeremy Pallas:** . **Mark Miller:** Writing – review & editing, Methodology, Investigation, Funding acquisition, Conceptualization. **Shaun Hicks:** Writing – review & editing, Investigation, Conceptualization. **Phillip Newton:** Writing – review & editing, Supervision. **Ginger Chu:** Writing – review & editing, Supervision. **John Paul Smiles:** Writing – review & editing, Conceptualization. **Michael Zhang:** Writing – review & editing, Methodology, Formal analysis.

## Ethics approval

Approved by the Hunter New England Human Research Ethics Committee – approval number 2023/ETH00202.

## Funding

This trial has been funded by a philanthropic grant from the Jack Murphy Memorial Society (since renamed as Jack’s Irish Society) which was administered by the Hunter Medical Research Institute (HMRI). Neither group had input into the development or conduct of this trial.

For part of the duration of this trial, the Principal Investigator (Jeremy Pallas) was supported by the Australian Governments Research Training Programme (RTP) Scholarship – to support a Higher Degree Research (HDR) programme.

## Declaration of competing interest

The authors declare that they have no known competing financial interests or personal relationships that could have appeared to influence the work reported in this paper.

## Figures and Tables

**Supplementary Figure 1:**
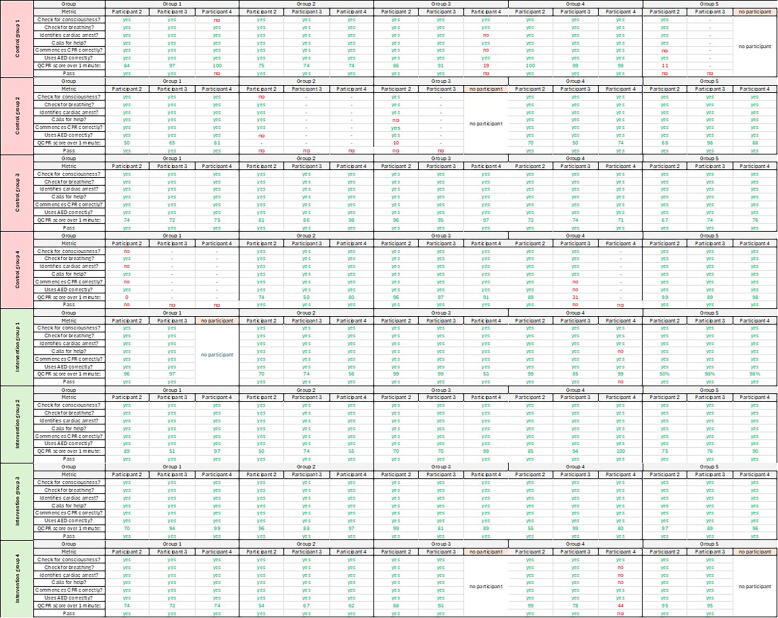
Full Trial Dataset
