# Supplementary material for: Exploration of ‘generational’ peer-led CPR training in the Australian community using blended learning approaches: a pilot randomised controlled trial
Source: Resusc Plus. 2025 Dec 15;27:101190. doi: 10.1016/j.resplu.2025.101190 (PMC12828363; doi:10.1016/j.resplu.2025.101190)
Supplement: Supplementary Data 1 [file mmc2.docx]

**Critical Item Checklist**

**Instructions:**

- An assessment space will be prepared with a Laerdal QCPR mannequin, a training AED and alcohol-based hand sanitiser.
- Participants will be introduced to the scenario outlined below and will be asked to talk through what they would do if they were the first responder to the scenario.
- The assessor will observe for the actions below to occur marking ‘yes’ or ‘no’ as applicable.
- When it comes time for CPR to be performed (as directed by the participant) the assessor will load the Laerdal QCPR instructor application and record the QCPR score achieved by the participant over 1 minute. Prior to this attempt the participant should be briefed on the process and asked to deliver their best attempt at CPR on the mannequin over the following 1 minute.
- While assessors may prompt for the next required step (asking ‘what would you do next’), there must be no direction or coaching provided on the expected answers.

**Scenario:**

You have walked into a room and found a middle 40-year-old man collapsed on the ground in front of you. This mannequin will represent the collapsed man. The environment appears safe and free of immediate danger. Could you please take me through the things that you would do as the first responder in this situation.

**To pass this assessment and progress to the next generation of training, a participant must perform all 6 core tasks appropriately as well as achieving over 50% as a QCPR score over 1 minute.**

**Stream**

| **Criteria** | **Generation 1** | **Generation 2** | **Generation 3** |
| --- | --- | --- | --- |
| 1. **Check for consciousness** |  |  |  |
| 1. **Check for breathing** |  |  |  |
| 1. **Identifies Cardiac arrest** |  |  |  |
| 1. **Calls for help (**including 000 call**)** |  |  |  |
| 1. **Commences CPR correctly** |  |  |  |
| 1. **Correctly uses an AED** |  |  |  |
| **Laerdal CPR score over 1 minutes (**%**)** |  |  |  |
| **Pass / Fail** |  |  |  |
