# Supplementary material for: Exploration of ‘generational’ peer-led CPR training in the Australian community using blended learning approaches: a pilot randomised controlled trial
Source: Resusc Plus. 2025 Dec 15;27:101190. doi: 10.1016/j.resplu.2025.101190 (PMC12828363; doi:10.1016/j.resplu.2025.101190)
Supplement: Supplementary Data 2 [file mmc3.docx]

| protocol  **The Chain of Survival Study** |
| --- |
| **Establishing the feasibility of peer-facilitated CPR education using a video-based training tool.** |
| Protocol Version 1.1 and date: *[Version 1.1; 01-03-2024]*  **Revision Chronology:**   \| **Date of change** \| **Summary of changes** \| \| --- \| --- \| \|  \| *Version 1; original document* \| \| **1-3-24** \| *Version 1.1: amended inclusion criteria* \| |
|  |
| **CONFIDENTIAL**  This document is confidential and the property of the Hunter Heart Safe. No part of it may be transmitted, reproduced, published, or used without prior written authorisation from the institution.  **Statement of Compliance**  This document is a protocol for a research project. This study will be conducted in compliance with all stipulation of this protocol, the conditions of the ethics committee approval, the NHMRC National Statement on Ethical Conduct in Human Research (2007) and the Note for Guidance on Good Clinical Practice (CPMP/ICH-135/95). |

Contents

[Investigator Roles and Responsibilities 2](#_Toc116033304)

[Background 2](#_Toc116033305)

[Literature Review 3](#_Toc116033306)

[Hypothesis 4](#_Toc116033307)

[Trial Design 5](#_Toc116033308)

[Study Setting 7](#_Toc116033309)

[Participants 7](#_Toc116033310)

[Outcomes 8](#_Toc116033311)

[Data Sources / Measurements 8](#_Toc116033312)

[Bias 8](#_Toc116033313)

[Study Size 8](#_Toc116033314)

[Statistical Methods 9](#_Toc116033315)

[Randomisation 9](#_Toc116033316)

[Data Management 9](#_Toc116033317)

[Harms 9](#_Toc116033318)

[Auditing 10](#_Toc116033319)

[Research Ethics Approval 10](#_Toc116033320)

[Consent or Assent 10](#_Toc116033321)

[Confidentiality 10](#_Toc116033322)

[Declaration of Interests 10](#_Toc116033323)

[Access to Data 11](#_Toc116033324)

[Ancillary and Post-Trial Care 11](#_Toc116033325)

[Dissemination Policy 11](#_Toc116033326)

[APPENDIX A – Participant Consent Form 12](#_Toc116033327)

[APPENDIX B 13](#_Toc116033329)

[APPENDIX C – Critical Item Checklist 16](#_Toc116033330)

[APPENDIX D – Laerdal QCPR Score Explained 18](#_Toc116033331)

[APPENDIX E – Email Invitation 20](#_Toc116033332)

[APPENDIX H – Email invitation (individual) 21](#_Toc116033334)

# Funding

This trial has been funded by a philanthropic grant from the Jack Murphy Memorial Society which is administered by the Hunter Medical Research Institute.

# Investigator Roles and Responsibilities

**Jeremy Pallas**

Nurse Educator – John Hunter Emergency Department

Phone: 0432035171

Email: [Jeremy.pallas@health.nsw.gov.au](mailto:Jeremy.pallas@health.nsw.gov.au)

Roles: Principle Investigator, study design and coordination, data collection, study team coordination, ethics submission, authorship.

**Dr Michael Zhang**

Staff Specialist – Emergency medicine (John Hunter)

Email: [michael.zhang@health.nsw.gov.au](mailto:michael.zhang@health.nsw.gov.au)

Roles: Study design and coordination, data collection, medical liaison, authorship.

**Dr John Paul Smiles**

Staff Specialist – Emergency medicine (John Hunter)

Email: [johnpaul.smiles@health.nsw.gov.au](mailto:johnpaul.smiles@health.nsw.gov.au)

Roles: Study design and coordination, data collection, medical liaison, authorship.

**Dr Mark Miller**

Staff Specialist – Emergency medicine (John Hunter)

Email: [mark.miller@health.nsw.gov.au](mailto:mark.miller@health.nsw.gov.au)

Roles: Study design, authorship.

**Shaun Hicks**

Clinical Nurse Specialist – Emergency medicine (John Hunter)

Email: [shaun.hicks@health.nsw.gov.au](mailto:shaun.hicks@health.nsw.gov.au)

Roles: Data collection, authorship.

# Background

Cardiopulmonary resuscitation is a skill that is widely taught in the community with varying methodological approaches and varying degrees of success. Despite the concept of lay person CPR training being many well established, the rates of attempted rescue by bystanders in the community remains unacceptably low (*CPR only attempted in 35% of out of hospital cardiac arrests prior to ambulance arrival as per the 2017 NSW Ambulance Service Cardiac Arrest Registry Data*).

Many systems around the world have significantly better uptake of CPR as a lay-person skill with CPR rates doubling our own and, in some cases, survival rates nearly tripling those found in Australia. World leading cardiac arrest response communities such as Seattle and Sweden are implementing new and innovative methods of disseminating CPR training to the wider community, alongside other novel methods of managing the initial resuscitation of a person suffering a cardiac arrest.

It is widely understood that the single most influential factor in whether a person recovers from a cardiac arrest that is within our control is whether or not they receive early CPR. For this to happen in more than 35% of the cases of cardiac arrest in the community, a rapid deployment of community targeted CPR training must be undertaken at a large scale.

An important part of understanding how to best disseminate this message widely is understanding the factors that influence successful CPR teaching to lay people.

# Literature Review

Survival rates for out of hospital cardiac arrest with survival to discharge are low and vary widely depending on global region (3.0% to 9.7%). The extensive geographical variation and poor outcomes associated with OOHCA have remained mostly static in the last three decades (1).

Some regions have worked on optimising the local chain of survival and have reported rates of survival to hospital discharge as high as 40%. The two factors that had the biggest impact on survival were early, good quality, CPR and early defibrillation (2,3). The importance of good quality bystander CPR initiated early in the resuscitation of patients cannot be emphasised enough and the rate of neurologically intact survival to discharge is directly related to this one factor (1, 4).

Internationally Norway (25%) (5), Seattle (21%) (6) and the Netherlands (21%) (7) lead the way with survival of OOHCA. The success in these countries has been attributed to early CPR and defibrillation made accessible through a system wide approach. In Norway, where survival is best with one in four cardiac arrests resulting in a favourable outcome, CPR training has been part of the national school curriculum since 1961.

Bystander CPR has also been closely linked with favourable outcomes, and areas with low socioeconomic status have been shown to have less bystander CPR and therefore reduced survival (1).

In May 2017, 2084 UK adults took part in a survey to help us discover how many people have trained in CPR or PAD use and how willing they are to act if they witnessed an OHCA. One in 5 survey respondents said they had witnessed a cardiac arrest; 58% reported that they had received CPR training and 22% PAD training. Training made a difference in people’s willingness to act. 74% of those trained in PAD use compared to 24% of those untrained said they would be willing to use one. For those trained in CPR, 76% were likely to perform CPR compared to 28% who were untrained. There is potential to increase survival rates through continuing attempts to improve public awareness and uptake of resuscitation skills training (8).

Within Australia, survival to discharge for OOHCA is 7.6% (1). Over the past year a group of local Doctors and Nurses from the Hunter Heart Safe Collaborative have tried to increase community awareness of cardiac arrest and how to manage it from the crucial first few seconds of its occurrence to the point trained ALS providers arrive to assist. This project has thus far, taught hands only CPR to over 2000 community members across the lower hunter region in a bid to improve rates of survival in OOHCA.

In their 2017 paper, Cartledge et al described the introduction of a video-based CPR instruction program into a cardiac rehabilitation group. This project was well received and was deemed effective in the evaluation, but one important finding of this trial was the willingness of the initial recipients of training to share the training with other. While the effectiveness of this secondary training was not assessed during this trial, this concept of peer facilitated training underpins the intervention described in this protocol.

# Aim

The primary aim of this study is to assess the comparative feasibility of a peer facilitated community CPR training model using either traditional face-to-face training or a hybrid training session based around a simple video-based training aid.

It is hypothesized that the efficacy of both initial training methods will be comparable on assessment. However, we anticipate that the introduction to shareable CPR training videos will be more successful in facilitating transfer of knowledge from one participant to the next – thus allowing the teaching to progress further with the aid than without.

# Trial Design

This study will utilise a prospective cohort design to evaluate the feasibility of peer facilitated CPR training provided by community members following either A) a traditional face to face CPR training session OR B) a hybrid model based around a simple video-based training aid.

Groups of 20 participants will be recruited to participate in this study through Hunter Heart Safe training sessions. These larger groups will then be further broken down into 5 ‘streams’ of 4 participants. The first participant in each of the streams (generation 1) will be designated to receive either traditional face to face CPR training OR a hybrid training session based around a simple video-based training aid. The participants will be randomized to either one of the two arms of training blocks based on a computer generated randomized sequence. The four participants following on from the initial trainee in each ‘stream’ are referred to as subsequent ‘generations’ i.e. the second person in a stream constitutes the second generation of that stream, the fourth person in sequence is the fourth generation etc.

Following on from their initial training, the first participants from each stream will then be tasked with teaching the lesson they have learned (one on one) to the second generation. If this message is conveyed successfully, the second generation will be tasked with teaching the third, and so on until either the message fails to be delivered appropriately, or all 4 generations within a single stream have been successfully taught CPR.

***
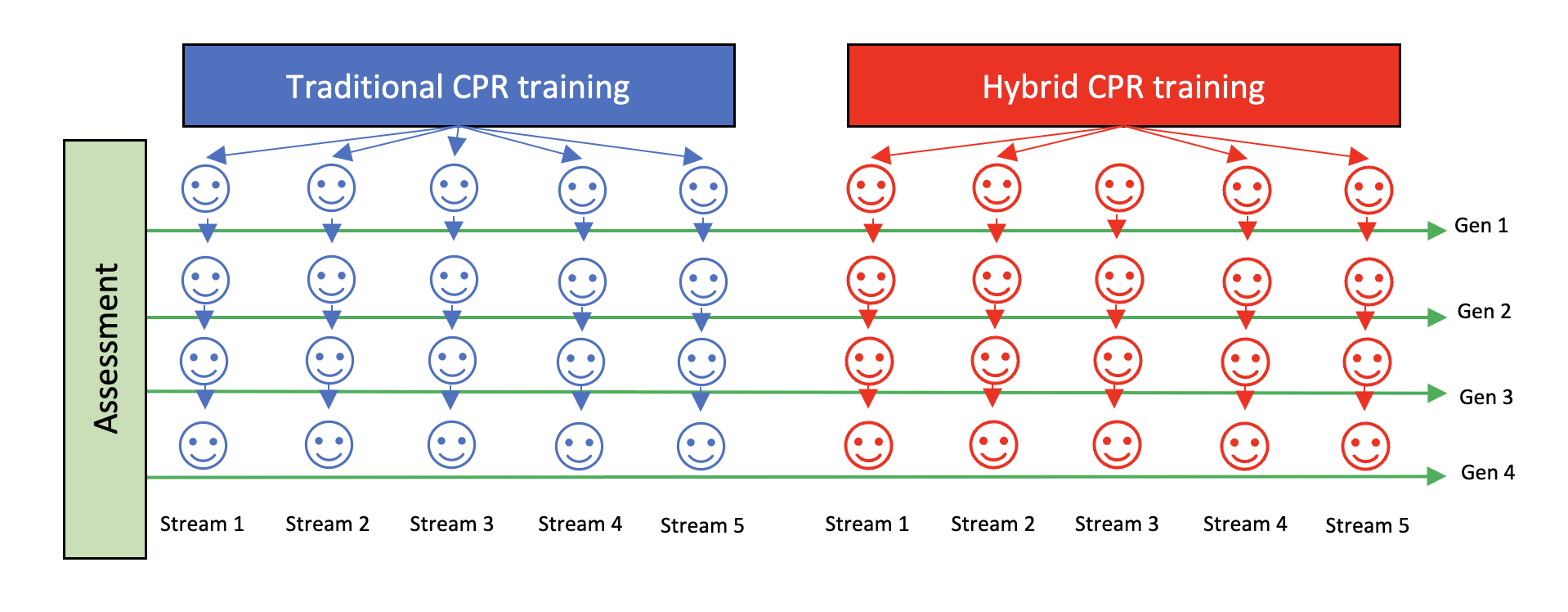
***

***Graphical Representation of the Study Design***

Following training of the initial participants, peer to peer training within each stream will be attended in separate rooms to avoid cross communication where possible. Once an individual has successfully delivered their training to the next generation in their stream, that person will have completed their participation in the trial. At the completion of the data collection period, a final large group session of conventional CPR training will be offered to all participants as a way of ensuring suitable validated training is offered to all participants.

For one generation to progress from learning to teaching, they must successfully complete an assessment against a ‘critical item checklist’ (as outlined in appendix C). The first generation will be excepted from this assessment as the initial the initial training sessions efficacy is not the primary focus of this trial. If the participant passes this assessment, they are instructed to teach the participant in the next generation of their stream what they have learned over a 20-minute period. If any member of the stream fails the assessment, data collection for that stream ends and all remaining members of that stream are deemed to have completed their participation in this trial. An assessment must be completed by every generation involved in peer facilitated teaching.’

# Study Setting

This study will be attended in the community using groups of participants volunteering to participate as a part of a scheduled community CPR training class delivered by the Hunter Heart Safe Group. Some sessions may be run in non-clinical areas within John Hunter Hospital or the Hunter Medical Research institute where suitable community locations cannot be sourced for the trainee group.

# Participants

In order to participate in this study, all participants must meet the following eligibility criteria:

- Age between 18-85 years
- No physical or medical limitation to performing chest compressions for 1 minute
- Must not have attended CPR training in the past 5 years

Participants for this study will be recruited from the pool of community members requesting to receive training from the Hunter Heart Safe group. Where recruitment to the trial is intended, groups will be approached with a recruitment email attached as appendix G. Along with the recruitment email, potential participant groups will be supplied with the participant information form attached below as appendix A.

In addition to participants from the general public, a number of participants may be sourced from the HMRI volunteer database. These volunteers will be engaged through the HMRI volunteer liaison through the delivery of the email invitation attached as appendix H along with the participant information form.

All participants taking place in this study will be provided with a $20 electronic gift card upon completion of their participation (i.e. at the end of the session).

The full timeline of each individual group session is outlined in the table below, noting that this example included the longest possible period required for participants to attend (assuming at least 1 stream makes it through 4 generations of assessment). Where no participants can progress to the next generation of training / assessment, the session will be completed early and where feasible the concluding training session may be brought forward in time to accommodate the needs of the participants.

| **Timeline for session** | **Activity** |
| --- | --- |
| **20 minutes (0:00-0:20)** | Introduction and consent process |
| **20 minutes (0:20-0:40)** | Initial Training for generation 1 (except in control group) |
| **20 minutes (0:40-1:00)** | Generation 1 attempt training of generation 2 |
| **20 minutes (1:00-1:20)** | Assessment of Generation 2 |
| **20 minutes (1:20-1:40)** | Successful Generation 2 attempt training of generation 3 |
| **20 minutes (1:40-2:00)** | Assessment of Generation 3 |
| **20 minutes (2:00-2:20)** | Successful Generation 3 attempt training of generation 4 |
| **20 minutes (2:20-2:40)** | Assessment of Generation 4 |
| **20 minutes (2:40-3:00)** | Final group training session (optional) |
| **Session concluded** | |

# Outcomes

The primary outcome being investigated in this study:

1. Can secondary learner driven peer to peer CPR training be delivered following an initial training session supported by CPR training videos:

- The primary measurement will be the raw number of successful CPR assessments (using the critical item checklist after a peer facilitated training episode) across all training groups.

The secondary outcome being investigated in this study:

1. Is a hybrid CPR training session using simple video-based training aids more effective than traditional face to face training:

- The primary measurement will be the comparative raw number of successful CPR assessments (using the critical item checklist after a peer facilitated training episode) between the face to face and hybrid CPR groups.

# Data Sources / Measurements

The following data sources will be used for assessment:

1. Can secondary learner driven peer to peer CPR training be delivered following an initial training session supported by CPR training videos:

- Assessment data will be collected from every participant following training. This assessment will utilise a critical item checklist (Appendix C), from which the participant must achieve each of the following goals to be deemed ‘successful’ in their assessment:
  - The participant must identify that the patient is unconscious and not breathing
  - The participant must call for help including a phone call to 000
  - The participant must commence CPR correctly (*2 hands on mid chest etc.*)
  - The participant must achieve a Laerdal ‘QCPR Performance Score’ of >50% over a 1-minute period (*as measured using the Laerdal resusci-annie mannequins using the adult compression only parameters*) – This scoring metric is explained in further detail in Appendix D.

# Study Size

A purposeful convenience sample will be employed for this trial owing to the feasibility nature and the contingent sequential recruitment model described. These factors together make it impractical for a formal sample size calculation to be attended.

This trial will intend to recruit 160 total participants across 8 groups of 20 possible participants with acknowledgement that the total number of recruited participants is dependent on the outcome of the serial assessments in each group. Recruitment will be shared equally between the traditional face to face CPR training group and the hybrid sessions based on the video training aid. Each group will be randomised to either the traditional face to face or hybrid groups electronically with the assignment being concealed in an opaque envelope which will be opened immediately prior to the commencement of the session.

While all efforts to maximise recruitment to ensure full groups of 20 will be taken, it is acknowledged that there may be attrition leaving some groups slightly short of the 20 participants. These details and reasons for the shortages will be reported along with the trial findings.

# Statistical Methods

The primary analysis method will follow the intention to treat principles. Differences between each of the 2 active cohorts (intervention arm, Hybrid CPR Training, and the control arm, Traditional CPR Training), will be assessed using a binomial test of proportions, with 95% confidence intervals for the difference presented together with the p value for the difference. A two-sided 90% confidence interval (equivalent to a 95% one sided interval) will be estimated for the difference in successful assessments between two different training teams. If the upper limit of this interval is < 0.15 the hybrid CPR Training arm will be declared non-inferior. Continuous secondary outcomes will be compared between groups using ANOVA models.

# Data Management

All data collected will be compiled via Redcap (*electronic survey software*). At the conclusion of the data collection period, the Redcap data will be exported in a spreadsheet which will then be stored on a central computer as well as a backup portable hard drive. Both the main study computer and backup hard drive will be password protected.

Hard copies of the critical item checklists used to mark the CPR assessments between generations will be scanned and stored on a central computer as well as a backup portable hard drive. The results of these checklists will also be added into a separate section of the spreadsheet containing the Redcap data for the purpose of data analysis.

# Harms

As only simulated patients will be used in this trial there will be no potential to cause harm to any living patient. The primary risk associated with this study is the potential for participants to receive substandard CPR training leading to a propagation of incorrect teaching within the wider community. Two measures are in place to control this risk:

1. Within each stream being examined, the progression of the peer-to-peer teaching will be ceased when a participant fails to achieve all items on the critical item checklist
2. At the end of each session, regardless of the group allocation, all groups will be offered standard group training delivered in a traditional face to face capacity by the Hunter Heart Safe Trainers (*this will be after all data has been collected so as to not confound the results of the study*)

# Auditing

As this is a small-scale trial in which the investigators will be directly overseeing the intervention and data collection there is no benefit in instituting procedures guiding the auditing of trial conduct.

# Research Ethics Approval

This trial will be submitted to the Hunter New England Human Research Ethics Committee for a relevant review.

# Consent or Assent

Informed consent will be gained from all participants before they partake in the trial. Members of the study group (*investigators*) will be responsible for ensuring that participants are adequately informed about the trial. This will involve a verbal briefing as well as the delivery of a participant information sheet to all prospective participants.

- A copy of the participant consent form used for this trial is attached as *appendix A*.
- A copy of the participant information form is attached as *appendix B*.

# Confidentiality

Any personal information collected during the course of this trial will be maintained in the confidence of the study group (*investigators*). No personal information will be shared outside of this group at any stage during the course of the trial.

Any clinical or practical data collected during the course of this trial will be maintained in the confidence of the study group while the project is ongoing. When the data are analysed and prepared for publication all personal identifiers will be removed.

# Declaration of Interests

The investigators involved in this trial are involved in the coordination of and training provided by the Hunter Heart Safe Group. This group is not for profit. There are no financial incentives or otherwise confounding interests to declare.

# Access to Data

The final study dataset will be available to all listed study contributors at completion of the data collection period. Upon completion of the data collection period, the data will be analysed and prepared for publication. Once the final data analysis is completed and the findings of this trial have been submitted for publication, the final trial data set will be made available to external parties upon request at the discretion of the principal investigator.

# Ancillary and Post-Trial Care

All participants will be able to contact the study group if additional information or support is required following their participation in this trial. All participants in this trial will be given a certificate of participation.

# Dissemination Policy

Following final analysis, the findings of this trial will be compiled and submitted for potential publication in a selected emergency medicine journal. All authors will collaboratively produce a final manuscript outlining the findings of this trial.

Once the final data analysis is completed and the findings of this trial have been submitted for publication, the final trial data set, protocol and statistical code used will be made available to external parties upon request at the discretion of the principal investigator.

**References:**

1. Aung Myat, Kyoung-Jun Song, Thomas Rea. Out-of-hospital cardiac arrest: current concepts. Lancet 2018; 391: 970–79
2. Becker L, Gold LS, Eisenberg M. et al. Ventricular fibrillation in King County, Washington: a 30-year perspective. Resuscitation 2008; 79: 22–27.
3. Bunch TJ, White RD, Gersh BJ, et al. Long-term outcomes of out-of-hospital cardiac arrest after successful early defibrillation. N Engl J Med 2003; 348: 2626–33
4. Sue Duval, PhD; Paul E. Pepe,MD, MPH; TomP. Aufderheide,MD, MS et al. Optimal Combination of Compression Rate and Depth During Cardiopulmonary Resuscitation for functionally Favorable Survival. JAMA Cardiology Published online August 14, 2019
5. 5. Lindner TW, Soreide E, Nilsen OB, et al. Good outcome in every fourth resuscitation attempt is achievable--an Utstein template report from the Stavanger region. Resuscitation 2011;82:1508-13.
6. Flemming D, Fogarty J. Public Health – Seattle and Kings County Division of Emergency Medical Services 2013 Annual Report to the King County Council. 2013
7. Berdowski J, Berg RA, Tijssen JG, Koster RW. Global incidences of out-of-hospital cardiac arrest and survival rates: Systematic review of 67 prospective studies. Resuscitation 2010;81:1479-87.
8. Claire A. Hawkes, PhD, RN; Terry P. Brown, PhD; Scott Booth et al. Attitudes to Cardiopulmonary Resuscitation and Defibrillator Use: A Survey of UK Adults in 2017. J Am Heart Assoc. 2019;8:e008267
9. Jin Hyuck Lee,1 Youngsuk Cho,1 Ku Hyun Kang,1. The Effect of the Duration of Basic Life Support Training on the Learners’ Cardiopulmonary and Automated External Defibrillator Skills. BioMed Research International Volume 2016.
